# Digital Twin-Enabled Dynamic Aggregation for Efficient Federated Learning

**DOI:** 10.3390/s26144460

**Published:** 2026-07-14

**Authors:** Wenqin Zhuang, Yuao Wang, Guocheng Wang

**Affiliations:** Jiangsu Key Laboratory of Intelligent Information Processing and Communication Technology, Nanjing University of Posts and Telecommunications, Nanjing 210003, China; wya@njupt.edu.cn (Y.W.);

**Keywords:** federated learning, client heterogeneity, digital twin, hierarchical aggregation, adaptive clustering

## Abstract

Federated learning (FL) enables collaborative model training without sharing raw data, but it faces challenges due to client heterogeneity, leading to inefficiency and reduced accuracy. This paper proposes a digital twin (DT)-based dynamic FL aggregation method to address these issues. The framework integrates a DT layer on the server side to perform preaggregation evaluations, simulating various aggregation strategies to select the optimal approach before actual global aggregation. An adaptive clustering method based on K-means is employed to group clients with similar characteristics, and a hierarchical aggregation evaluation strategy is designed to optimize both intra-cluster and inter-cluster aggregation, with the goal of minimizing latency and energy consumption while maximizing model accuracy. Simulation results on the MNIST and CIFAR-10 datasets demonstrate that the proposed method not only accelerates model convergence and improves accuracy but also significantly reduces training latency and energy consumption costs compared with baseline FL algorithms. This DT-assisted approach delivers a practical and effective optimization solution for federated learning deployment over large-scale heterogeneous IoT sensor networks.

## 1. Introduction

Federated learning (FL) is a promising distributed machine learning technique that supports privacy-preserving collaborative training, which fits well with intelligent perception and data analysis tasks in large-scale Internet of Things (IoT) and wireless sensor networks [1]. With the widespread deployment of intelligent sensing devices in industrial, urban, and edge scenarios, massive heterogeneous sensing data is continuously generated by distributed sensor nodes. As a privacy-friendly distributed training paradigm, FL avoids raw sensing data collection and transmission and enables global model optimization through terminal collaboration. First proposed by Google in 2016, FL distributes training tasks to multiple sensing clients. Each sensor-enabled edge device performs local training and uploads only model parameters, which are aggregated by the central server to update the global model. This framework supports multi-node collaborative intelligent sensing while protecting data privacy in sensor networks [2]. By securing privacy-sensitive sensing data and facilitating cross-device data collaboration, FL has become a fundamental enabler of artificial intelligence and intelligent perception applications in next-generation sensor networks [3].

Nevertheless, FL encounters notable bottlenecks in the model aggregation stage under dynamic sensor network conditions. The classic federated averaging (FedAvg) algorithm treats all clients equally and ignores the inherent heterogeneity of practical IoT sensor nodes, resulting in degraded training efficiency and model accuracy [4]. Real-world sensor clients differ greatly in data quality, sampling scale, computing capability, and communication resources [5,6]. In multi-sensor collaborative perception scenarios, inconsistent data distribution and reliability across heterogeneous IoT terminals make simple average aggregation ineffective. Such static aggregation easily causes slow convergence and poor generalization, restricting the stable deployment of FL-based intelligent sensing in large-scale sensor networks [7,8].

Extensive research on model aggregation has been conducted both domestically and internationally. By adjusting aggregation weights according to client data characteristics and training status, the convergence speed and accuracy of the model can be improved [9,10]. Bensiah et al. [11] proposed a weighted aggregation method based on the data distributions of clients, assigning larger weights to clients with higher-quality data to enhance model performance. In [12], the authors addressed the challenges posed by attacks on FL to data privacy and security and proposed a FL-secure aggregation method that hides weights to mitigate the risk of inference attacks caused by weight leakage during FL aggregation. The heterogeneous data distribution across IoT devices significantly slows the convergence speed of the global model and may even lead to a significant reduction in model accuracy [13,14].

To address this issue, the authors in [15] developed a coefficient-of-variation-based federated adaptive aggregation algorithm, which adaptively adjusts the aggregation weight factors of local client models according to the difference in the coefficient of variation obtained before and after local model training. Additionally, the proportion of the current global model during the aggregation process is dynamically adjusted in the middle to late stages. In [16], the authors proposed an efficient communication-based federated aggregation algorithm named FedSL to reduce communication overhead. On the basis of the number of global model layers, FedSL divides client models into multiple groups in the depth dimension and adopts a Max-Min client selection strategy to select participants for each layer. Each client transmits only a subset of the parameters for the selected layer, thus reducing the number of parameters. FedSL aggregates the global models within each group and connects all the group parameters in layer order. In [17], the authors introduced a novel FL aggregation method to address data heterogeneity issues. First, a composite similarity-based weighting mechanism is employed, which integrates cosine similarity and Gaussian similarity measures to dynamically optimize client contributions. Second, an approximation term is incorporated into the client weighting scheme, prioritizing updates that are closer to the global optimum based on gradient norms, which improves model convergence and robustness. Finally, a dynamic parameter learning technique is introduced to adjust the balance between similarity metrics according to data heterogeneity, thus optimizing the aggregation process.

However, adopting a fixed model aggregation method makes adapting to the dynamic changes in client computing power and network status difficult, and such a method also fails to fully exploit the potential connections among clients. In FL environment with a highly heterogeneous data distribution, local models trained by different clients may vary substantially, and using different aggregation methods can exacerbate performance disparities, which leads to slow model convergence and reduced accuracy [18,19]. Notably, flexibly selecting the model aggregation method in each iteration of training still poses a severe challenge, especially in dynamically changing scenarios.

Digital twins (DT) is an emerging virtual‒physical interaction technology that can simulate and predict the operating status of a system in real time by constructing a virtual mirror image of a physical entity [20], thus providing a new solution to the model aggregation problem [21,22]. Recent studies have further shown that DT has been increasingly integrated with machine learning and intelligent systems. For example, DT has been discussed as an important enabling technology for embodied artificial intelligence, where virtual–physical interaction can support perception, decision-making, and autonomous learning [23]. In industrial scenarios, DT has also been combined with deep transfer learning for equipment fault prediction, such as gearbox fault diagnosis and health monitoring [24]. These studies indicate that DT is evolving from system-state mirroring toward intelligent prediction, decision support, and learning-enhanced optimization. By using DT models to monitor the data distribution and training status of clients in real time, the aggregation weights can be dynamically adjusted to optimize the global model updating process [25]. This method can effectively address client data heterogeneity and dynamic changes and improve the overall performance of FL. Additionally, DT technology can introduce virtual clients to simulate different data distributions and training scenarios, providing more diverse and abundant training data for model aggregation [26].

Extensive research has demonstrated the effectiveness of digital twins in enhancing federated learning decision-making. By creating digital representations of physical systems, DT formulates optimal decisions, consequently improving federated learning performance [27,28,29]. Lu et al. proposed the digital twin edge networks (DITENs). By employing an asynchronous model update scheme, communication efficiency was significantly enhanced while transmission energy consumption costs were effectively reduced [27]. Guo et al. proposed a trust evaluation scheme for federated learning-assisted digital twins for mobile networks (DTMN), designing a user behavior model based on multi-attribute features to further enhance system reliability [28]. Tang et al. proposed a digital twin (DT) and blockchain-assisted federated learning (FL) framework. In addition, a blockchain-based verification mechanism and a validator selection algorithm have been designed to ensure the integrity of the model and the authentication of participants during the FL process [29]. Sun et al. utilized digital twins to capture the characteristics of industrial equipment to support federated learning. Considering potential deviations arising from digital twins, they proposed a credibility-based aggregation method for federated learning to mitigate the impact of such deviations [30].

Although existing works demonstrate the potential of DT-enhanced FL for sensing systems, current DT applications mainly focus on state monitoring, trust evaluation, and communication optimization. Few studies concentrate on DT-driven preaggregation virtual evaluation and dynamic aggregation optimization for sensor networks. Most prior methods cannot quantitatively predict the latency, energy consumption, and accuracy of different aggregation schemes before physical aggregation, limiting the dynamic optimization capability in time-varying IoT environments. Moreover, traditional FL optimizations, including client selection, weighted aggregation, and clustered FL, rely on fixed rules or real-time observable states. Different from these methods, this work constructs a dedicated DT virtual evaluation layer for heterogeneous sensor networks. Before real server-side aggregation, multiple intra-cluster and inter-cluster aggregation strategies are comprehensively simulated and evaluated. The optimal scheme is dynamically selected to achieve adaptive optimization of both sensing client participation and aggregation strategies for complex IoT sensing scenarios.

To further clarify the difference between the proposed framework and existing methods, Table 1 provides a qualitative comparison from the perspectives of decision basis, client clustering, hierarchical aggregation, DT-based evaluation, and dynamic aggregation strategy selection.

Motivated by these observations, the principal scientific novelty of this work lies in the DT-driven preaggregation evaluation mechanism. Different from conventional FL aggregation methods that directly execute aggregation according to fixed rules or observed client states, the proposed method uses the DT layer to virtually evaluate candidate aggregation strategies before physical aggregation. The adaptive client clustering and hierarchical aggregation strategy are designed as supporting mechanisms to reduce the strategy evaluation space and organize the aggregation process at both intra-cluster and inter-cluster levels. Therefore, the overall contribution of this work is an integrated DT-based optimization framework that jointly considers client state mapping, client clustering, virtual strategy evaluation, and physical-layer aggregation execution for dynamic FL aggregation. Specifically, the main contributions are summarized as follows.

(1) We propose a DT-based preaggregation evaluation framework for dynamic FL aggregation. Different from conventional client selection or weighted aggregation methods that directly make decisions based on observed client states, the proposed framework constructs a DT layer on the server side to perform preaggregation evaluation before actual physical aggregation. The DT layer virtually evaluates candidate aggregation strategies and provides aggregation decisions for the physical FL process, thereby improving the adaptability of FL under heterogeneous and dynamic client conditions.
(2) We design a client-adaptive clustering and hierarchical aggregation evaluation strategy in the DT layer. Unlike existing clustered FL methods that mainly perform aggregation based on fixed cluster-level rules, the proposed method evaluates both intra-cluster and inter-cluster aggregation strategies. In this way, the participating clients and aggregation methods can be dynamically selected according to latency, energy consumption, and model accuracy.
(3) We conduct extensive simulations to validate the effectiveness of the proposed framework in heterogeneous data environments. By comparing our approach with multiple baseline FL algorithms, the comprehensive results demonstrate that our scheme significantly reduces system costs in terms of communication latency and energy consumption while maintaining superior model accuracy, effectively enhancing the overall intelligence and flexibility of the FL system.

## 2. System Model

**Figure 1:**
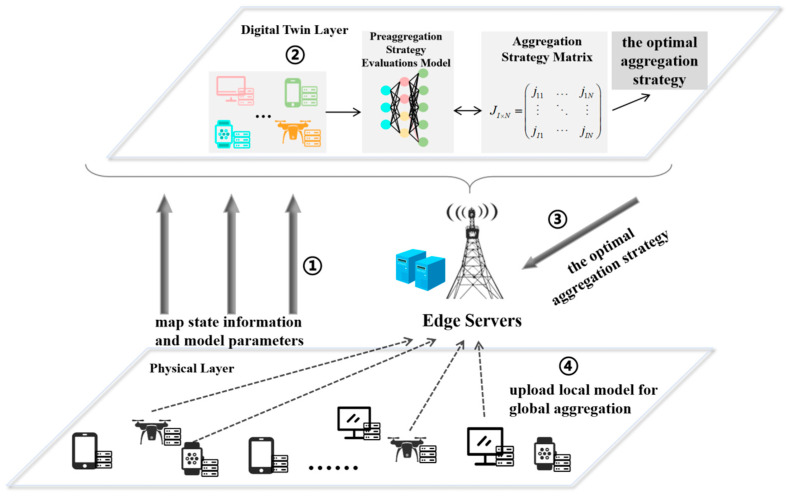
Dynamic aggregation architecture for federated learning models.

The system model is illustrated in Figure 1, which is specifically divided into a physical layer and a digital twin layer. The physical layer consists of one edge server and multiple clients. The set of clients is defined as $N=\left\{1,2,\ldots ,n,\ldots ,N\right\}$, and their dataset is denoted as $D_{n}$, which is of size $\left|D_{n}\right|$. The digital twin layer is constructed on the edge servers to leverage their higher computational power to simulate preaggregation strategy evaluations. In each iteration, clients train local models on their local datasets and map their state information $x_{n}$ and local model parameters $\omega_{n}$ to the DT layer for preaggregation evaluation. The DT layer extracts features $\tilde{x_{n}},\tilde{\omega_{n}}$ from client information and then trains a preaggregation model using these features to evaluate various model aggregation strategies. Finally, the edge server aggregates the local models of the clients based on the optimal aggregation strategy provided by the DT layer, generates a new global model, and distributes it to clients for the next training round. This layered model architecture enables efficient preaggregation and dynamic optimization of client models, thereby significantly increasing training efficiency and accuracy.

### 2.1. Communication Model

We assume that B is the total uplink bandwidth provided by the edge server, which is evenly distributed among the clients. During iteration k, the uplink rate for clients transmitting parameters to the edge server is [33]

(1) $$r_{n}^{up,k}=\frac{B}{N}log_{2}\left(1+\frac{p_{u}^{k}{\left(h_{u}^{k}\right)}^{2}}{N_{0}}\right),$$

where B denotes the total uplink bandwidth, $p_{u}^{k}$ represents the client’s transmission power, $N_{0}$ represents the variance in the Gaussian channel white noise, $h_{u}^{k}=o_{n}d_{n}^{-2}$ indicates the channel gain between the client and the edge server, $d_{n}$ represents the distance between the client and the edge server, and $o_{n}$ represents the Rayleigh fading parameter. Under the assumption that the bandwidth allocated by the server to the client remains constant, the downlink transmission rate for the server broadcasting updated global model parameters to the client is as follows:

(2) $$r_{n}^{down,k}=\frac{B}{N}log_{2}\left(1+\frac{p_{n}^{k}{\left(h_{n}^{k}\right)}^{2}}{N_{0}}\right),$$

where $p_{n}^{k}$ denotes the server’s transmission power and $h_{n}^{k}$ denotes the channel gain.

The communication model has three components: transmission latency for the client uploading local model parameters, transmission latency for the server broadcasting the global model, and transmission energy consumption for the client uploading local model parameters.

(1) Uplink communication latency: After receiving the optimal aggregation strategy from the DT layer, selected clients upload their local model parameters to the server for global aggregation. Under the assumption that the number of uploaded model parameters is $l_{n}^{up,k}$ and that all the clients have identical local model parameters, the uplink transmission latency from the clients to the server is $T_{n}^{up,k}=l_{n}^{up,k}/r_{n}^{up,k}$.
(2) Downlink communication latency: The server performs global parameter aggregation to obtain the updated global model and broadcasts the updated global model parameters to all the clients [31]. We let the number of broadcast global model parameters be $l_{n}^{down,k}$, and we assume that they are identical. The downlink transmission latency for the server broadcasting the global model parameters to clients is $T_{n}^{down,k}=l_{n}^{down,k}/r_{n}^{down,k}$. Therefore, in the k-th iteration round, the total communication latency is $T_{n}^{tran,k}=T_{n}^{up,k}+T_{n}^{down,k}$.
(3) Uplink transmission energy consumption: The time required for clients to transmit local model parameters to the server is denoted as $t_{n}^{up,k}=l_{n}^{up,k}/r_{n}^{up,k}$. Consequently, the uplink transmission energy consumption is expressed as follows:
  (3) $$E_{n}^{tran,k}=p_{u}^{k}\cdot t_{n}^{up,k}=\frac{p_{u}^{k}\cdot l_{n}^{up,k}\cdot N}{Blog_{2}\left(1+\frac{p_{u}^{k}{\left(h_{u}^{k}\right)}^{2}}{N_{0}}\right)}.$$

### 2.2. Computational Model

(1) Local computation latency

Clients perform local update training on the basis of the local dataset $D_{n}$. The energy consumption of local computing depends on the size of the dataset and the number of CPU cycles processed per second. We let $c_{n}$ denote the number of CPU cycles required by the client to process one data sample. The total number of cycles for one local iteration is $c_{n}\cdot \left|D_{n}^{k}\right|$. During iteration round k, the CPU cycle frequency allocated to local computation is denoted as $f^{k}$. Under the assumption that the client’s computational capacity is divided into J tiers, corresponding CPU cycle frequencies $f^{k}\in \left\{F_{1},F_{2},\ldots ,F_{J}\right\},f^{k}\leq F^{max}$ are assigned on the basis of the tiering results, where $F^{max}$ represents the maximum available computational resources on the client. The computational latency for the client during iteration k is as follows:

(4) $$T_{n}^{cmp,k}=\frac{c_{n}\cdot \left|D_{n}^{k}\right|}{f^{k}}.$$

(2) Local computational energy consumption

During iteration k, the client’s computational energy consumption is expressed as follows [34]:

(5) $$E_{n}^{cmp,k}=\frac{\partial }{2}\cdot {\left(f^{k}\right)}^{2}\cdot c_{n}\left|D_{n}^{k}\right|,$$

where $\frac{\partial }{2}$ denotes the effective capacitance coefficient of the computational chipset.

(3) Model aggregation latency

Model aggregation latency refers to the time consumed by the server during global model aggregation in a given iteration round of training and encompasses both the twin-layer preaggregation evaluation latency and the server aggregation latency. The aggregation latency depends on the number of model parameters, computational complexity, and complexity of the aggregation algorithm:

(6) $$T_{agg}^{k}=G_{pre}(M,C_{DT})+G_{agg}(M,C,A),$$

where M denotes the number of model parameters, $C_{DT},C$ represent the computational complexity of DT model prediction and server aggregation, respectively, and A indicates the complexity of the aggregation algorithm. In practice, the server aggregation latency for a given iteration is measured as the time difference between the start and end of training.

(4) Model aggregation energy consumption

The model aggregation energy consumption refers to the energy that is expended by the server during global model aggregation and encompasses both the energy used for preaggregation evaluation at the DT layer and the energy consumed during server-side aggregation. The aggregation energy consumption is directly related to the server’s power consumption and aggregation latency:

(7) $$E_{agg}^{k}=P_{s}T_{agg}^{k},$$

where $P_{s}$ represents the power consumption of the server.

### 2.3. Problem Formulation

The proposed federated learning dynamic aggregation method based on digital twins simulates various aggregation strategies at the DT layer to evaluate their performance and outputs the optimal aggregation strategy. This includes the list of participating clients and the aggregation method, with the aim of minimizing the total latency and energy consumption of FL model training. In iteration round k, the set of clients selected for aggregation is denoted as $S^{k}$. For clients, both communication latency and computation latency are considered in this study. The client latency in iteration round k is defined as $T_{n}^{k}=T_{n}^{tran,k}+T_{n}^{cmp,k}$, which is determined by the last client in set $S^{k}$ to complete its upload. Thus, the iteration latency for round k is the sum of the client latency and aggregation latency:

(8) $$T^{k}=\underset{n\in S^{k}}{max}T_{n}^{k}+T_{agg}^{k}.$$

During training iteration k, the client energy consumption comprises the communication energy $E_{n}^{k}=E_{n}^{tran,k}+E_{n}^{cmp,k}$ and computational energy. The total training energy consumption is the sum of the energy from all participating clients and the aggregation energy. Thus, the iteration energy for training iteration k is:

(9) $$E^{k}=\sum_{n\in S^{k}}E_{n}^{k}+E_{agg}^{k}$$

After K rounds of iteration, the total latency of the server is $T_{total}=\sum_{k=1}^{K}T^{k}\left(p_{n}^{k},l_{n}^{up,k},l_{n}^{down,k}\right)$, and the total energy consumption of the clients is $E_{total}=\sum_{k=1}^{K}E^{k}(p_{u}^{k},f^{k})$. In this paper, we proposed a mixed-integer nonlinear optimization problem.

The objective is to select a subset of clients to participate in aggregation and to determine the model aggregation method for each round, such that the total latency and total energy consumption after K iterations are minimized.

The objective function is as follows:

(10) $$\begin{array}{l} P1:\underset{K\in \left\{1,2,3\ldots \right\}}{min}\sum_{k=1}^{K}\sum_{n\in S^{k}}\left[\xi \underset{n\in S^{k}}{max}T_{n}^{k}+(1-\xi )E_{n}^{k}\right]\\ s.t.\;C_{1}:f^{k}\in \left\{F_{1},F_{2},\ldots ,F_{J}\right\},f^{k}\leq F^{max}\\ \;\;\;\;\;\;\;C_{2}:S^{k}\in N\&\left|S^{k}\right|>0\\ \;\;\;\;\;\;\;C_{3}:B\leq \lambda_{B}\&B/N>0\\ \;\;\;\;\;\;\;C_{4}:E_{n}^{k}\leq \lambda_{E}\\ \;\;\;\;\;\;\;C_{5}:\omega^{k}\geq \omega^{*} \end{array},$$

where ξ represents a weighting factor that adjusts the relative importance of the communication latency and computational energy consumption. In problem (P1), latency and energy consumption are two cost terms to be minimized. Before calculating the weighted objective, the latency and energy terms are normalized to avoid the scale imbalance caused by different physical units. The coefficient ξ controls the trade-off between latency and energy consumption. A larger ξ gives higher priority to latency reduction, whereas a smaller ξ emphasizes energy saving. In the experiments, ξ is set to 0.5 to balance latency and energy consumption unless otherwise specified. Constraint C1 limits the CPU frequency allocated to the clients should be less than the maximum available computational resources and greater than the minimum computational resources that the server can provide. Constraint C2 limits the number of the clients selected for aggregation should be less than the total number of clients but greater than zero. Constraint limits the total bandwidth B allocated to all clients should be less than the maxim C3 μm bandwidth setting $\lambda_{B}$. Constraint C4 limits the total energy consumption of clients during training round k to be below the upper bound. Constraint C5 requires that the model accuracy $\omega^{k}$ achieved in each iterative training round reach the preset model accuracy $\omega^{*}$.

## 3. A Digital Twin-Based Dynamic Aggregation Algorithm for Federated Learning

In this section, we propose the DT-FL algorithm, a digital twin-based federated learning dynamic aggregation approach. It incorporates a client-side adaptive clustering method and a hierarchical aggregation evaluation strategy, both of which are implemented in the DT layer. Within the DT layer of FL, the adaptive clustering method dynamically groups clients using clustering algorithms. A hierarchical aggregation evaluation strategy is then used to assess and aggregate models within each cluster. Finally, the optimal aggregation strategy is selected to form the global model.

**Figure 2:**
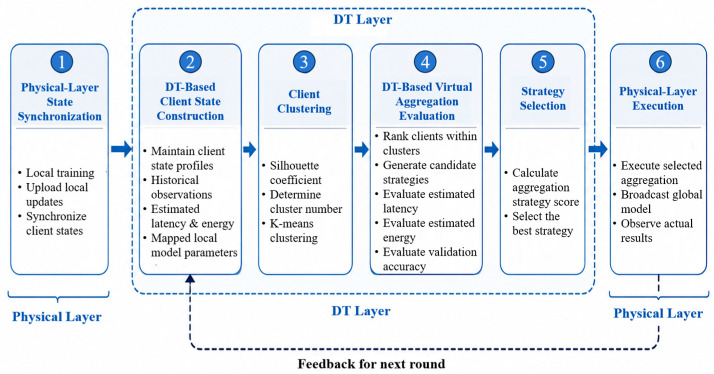
Overall workflow of the proposed DT-FL framework.

To further clarify the sequence of operations in the proposed framework, Figure 2 presents the overall workflow of DT-FL. Specifically, the physical-layer client states are first synchronized to the DT layer, where client state profiles are constructed and updated. Then, the DT layer performs client clustering and virtual aggregation evaluation to compare candidate aggregation strategies in terms of latency, energy consumption, and validation accuracy. Based on the evaluation results, the best aggregation strategy is selected and executed in the physical layer. Finally, the observed execution results are fed back to the DT layer to support the next training round.

### 3.1. K-Means-Based Client-Adaptive Clustering Method

The K-means-based client-adaptive dynamic clustering method proposed in this section is implemented in the DT layer. By constructing a virtualized client feature space within the DT layer to characterize the resource and performance profiles of physical-layer devices, the DT model can accurately quantify the impact of cluster granularity on global training latency and energy consumption. This predictive analysis enables the optimization of system configurations without incurring the overhead of actual physical-layer resources. First, by leveraging the high-fidelity simulation capabilities of the DT, the optimal number of clusters I is determined via the silhouette coefficient method. Subsequently, the DT layer executes the clustering algorithm to construct an effective FL logical topology. Finally, a DT-driven hierarchical evaluation strategy is employed to pre-assess the performance of various clusters, thereby deriving the optimal aggregation strategy to guide physical-layer execution.

(1) Determination of the Optimal Number of Clusters

Based on the system model in Section 2, the DT layer maintains a state profile for each physical client before clustering. The latency and energy features used for clustering are not the unknown optimization results of the current aggregation round. Instead, they are estimated client-level state features available in the DT layer before the aggregation decision is made. Specifically, $T_{n}^{k}$ denotes the estimated total latency of client n before aggregation in training round k, including communication latency and local computation latency. Similarly, $E_{n}^{k}$ denotes the estimated total energy consumption of client n before aggregation in training round k, including uplink transmission energy and local computation energy. These values are obtained according to the communication and computation models in Section 2 and are updated using the client status observed in previous completed training rounds.

For the first training round, the DT layer initializes the client state profile according to the initial system parameters, including the local dataset size, CPU frequency, transmission power, channel gain, and distance between the client and the edge server. After each completed training round, the DT layer updates the latency and energy features according to the observed communication latency, computation latency, transmission energy consumption, and computation energy consumption of each client. Therefore, the feature vector used for clustering in round k is constructed before the aggregation decision of that round and reflects the estimated or historical resource status of each client, rather than the final optimization result of the current round.

To maintain consistency with the optimization objective of reducing latency and energy consumption, the DT layer defines the client feature vector $x_{n}^{k}$ across three dimensions: local dataset size $\left|D_{n}\right|$, estimated total latency $T_{n}^{k}$, and estimated total energy consumption $E_{n}^{k}$. The feature vector is formulated as follows:

(11) $$x_{n}^{k}=\left\{\left|D_{n}\right|,T_{n}^{k},E_{n}^{k}\right\}$$

where $\left|D_{n}\right|$ reflects the potential data contribution of client n, while $T_{n}^{k}$ and $E_{n}^{k}$ describe the estimated resource cost of the client before aggregation in round k. Although latency and energy consumption are also included in the optimization objective, their use in clustering does not introduce circular logic. The clustering process uses DT-maintained prior state information to group clients with similar data-resource characteristics, whereas the optimization objective evaluates the latency and energy consumption resulting from the selected aggregation strategy. Therefore, clustering and strategy evaluation are sequentially related but not circular.

The silhouette coefficient is a metric for evaluating clustering quality; it aids in determining the optimal number of clusters. It assesses clustering effectiveness by measuring the compactness and separation of each sample. For each client, the silhouette coefficient $s_{n}$ is defined as follows:

(12) $$\begin{array}{l} s\left(n\right)=\frac{b(n)-a(n)}{max\left\{a(n),b(n)\right\}}\\ a(n)=\frac{1}{\left|C_{i}\right|-1}\sum_{m\in C_{i},m\neq n}d(n,m)\\ b(n)=\underset{C_{j}\neq C_{i}}{min}\left(\frac{1}{\left|C_{i}\right|}\sum_{j\in C_{j}}d\left(n,j\right)\right) \end{array},$$

where $s\left(n\right)$ ranges from −1 to 1. Values closer to 1 indicate more reasonable client clustering and better clustering quality. a(n) represents the intra-cluster distance within the cluster $\tilde{C_{i}}$ to which the client belongs, $\tilde{\left|C_{i}\right|}$ denotes the number of clients $\tilde{C_{i}}$ within this cluster, and d(n,m) represents the Euclidean distance between clients n and m. b(n) denotes the average distance from the client to the nearest cluster; a larger value indicates greater separation from other clusters and better clustering quality. $\tilde{C_{j}}$ represents all clusters except $\tilde{C_{i}}$, $\tilde{\left|C_{j}\right|}$ denotes the number of clients within cluster $\tilde{C_{j}}$, and $d\left(n,j\right)$ denotes the Euclidean distance between clients n and j. In this study, the Euclidean distance $\tilde{d(n,m)}$ between clients across the DT layer is calculated using the defined feature vector $\tilde{x_{n}}=\left\{\tilde{\left|D_{n}^{k}\right|},\tilde{T_{n}^{k}},\tilde{E_{n}^{k}}\right\}$:

(13) $$\tilde{d(n,m)}=\sqrt{{\left(\tilde{\left|D_{n}^{k}\right|}-\tilde{\left|D_{m}^{k}\right|}\right)}^{2}+{\left(\tilde{T_{n}^{k}}-\tilde{T_{m}^{k}}\right)}^{2}+{\left(\tilde{E_{n}^{k}}-\tilde{E_{m}^{k}}\right)}^{2}}.$$

To evaluate the clustering effectiveness across the entire client set, the average of the silhouette coefficients of all the clients is computed:

(14) $$s_{avg}=\frac{1}{N}\sum_{n=1}^{N}s\left(n\right),$$

where N denotes the total number of clients, and the average silhouette coefficient $s_{avg}$ ranges from −1 to 1, with a value approaching 1 signifying superior clustering quality. To identify the optimal topology, a heuristic search is conducted within the DT layer across the predefined set of cluster numbers $I=\left\{2,3,\ldots ,10,\ldots \right\}$. By iterating through each value of I, the system executes the K-means algorithm in the DT layer to simulate potential clustering outcomes. Subsequently, the DT layer calculates the individual silhouette coefficient s(n) for each client and the corresponding global average $s_{avg}$. Based on a comprehensive evaluation of these simulation results, the DT identifies the specific I that maximizes $s_{avg}$ as the optimal cluster count, thereby establishing a strategic foundation for physical-layer execution.

(2) DT-Driven K-Means Clustering Algorithm

**Figure 3:**
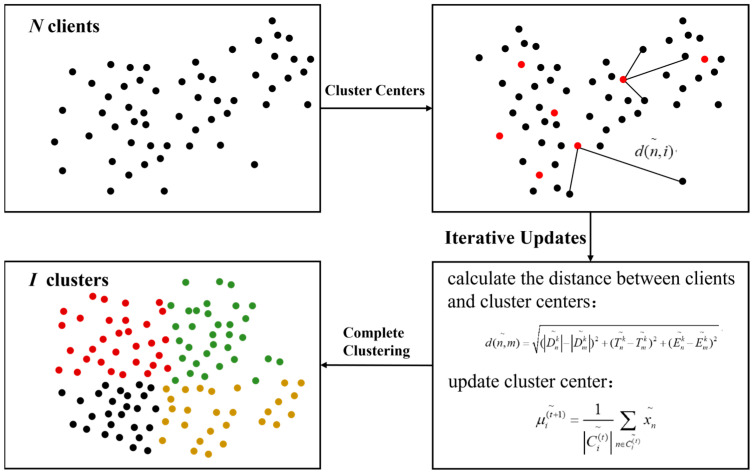
K-Means Clustering Algorithm Diagram.

Clustering is the process of dividing a given set of objects into multiple disjoint clusters on the basis of predefined criteria such that objects within the same cluster are more similar than those in different clusters are [32,34,35]. As shown in Figure 3, within the DT-driven clustering algorithm, the DT layer N matches each physical client with the corresponding I cluster centers.

The clustering steps are as follows:

(1) Cluster centers are initialized by randomly selecting feature vectors $\tilde{x_{n}}$ from the synchronized DT profiles of the clients I:

(15) $$\tilde{\mu }=\left\{\tilde{\mu_{1}^{\left(0\right)}},\tilde{\mu_{2}^{\left(0\right)}},\ldots ,\tilde{\mu_{i}^{\left(0\right)}},,\ldots ,\tilde{\mu_{I}^{\left(0\right)}},\right\},$$

where $\tilde{\mu_{i}^{\left(0\right)}}$ denotes the initial center of cluster i in the DT layer.

To optimize computational efficiency, the Euclidean distances between clients are precomputed. Afterward, an N×I matrix A is constructed in the DT layer, as shown in Table 1, where each element $A\left(n,i\right)$ represents the distance between the corresponding client and cluster center. The minimum nonzero Euclidean distance $\tilde{d_{\min}(n,i)}$ between a client and all the cluster centers across N distinct rows and I distinct columns in A is searched, and the client is assigned to that cluster.

(2) To optimize computational efficiency, the Euclidean distances between clients and cluster centers are precomputed based on the state information mapped to the DT. Afterward, an N×I matrix A is constructed in the DT layer, as shown in Table 2, where each element $A\left(n,i\right)$ represents the distance between the corresponding client and a cluster center. The minimum non-zero Euclidean distance $\tilde{d_{\min}(n,i)}$ between a client and all cluster centers across N distinct rows and I distinct columns in A is searched in the DT layer, and the client is assigned to that cluster.

(3) The mean of the feature vectors for all clients assigned to each cluster is computed within the DT layer, and the cluster center is updated accordingly:

(16) $$\tilde{\mu_{i}^{\left(t+1\right)}}=\frac{1}{\tilde{\left|C_{i}^{\left(t\right)}\right|}}\sum_{n\in \tilde{C_{i}^{\left(t\right)}}}\tilde{x_{n}},$$

where $\tilde{\left|C_{i}^{\left(t\right)}\right|}$ denotes the number of clients in cluster B and t represents the iteration count for updating cluster centers during clustering.

(4) Steps (2) and (3) are repeated until the change in all the cluster centers falls below a preset threshold or the preset iteration count κ is reached, thus indicating algorithm convergence and termination (Algorithm 1), respectively.
**Algorithm 1** K-Means-Based Client Clustering Algorithm$\mathrm{Input}:\;\mathrm{Feature}\;\mathrm{vector}\;\tilde{x_{n}}=\left\{\tilde{\left|D_{n}^{k}\right|},\tilde{T_{n}^{k}},\tilde{E_{n}^{k}}\right\}$, number of clusters I, total number of clients N, maximum number of iterations κ, convergence threshold ε.Output:
I
$\;\mathrm{client}\;\mathrm{clusters}\;\tilde{C}=\left\{\tilde{C_{1}},\tilde{C_{2}},\ldots ,\tilde{C_{i}},\ldots ,\tilde{C_{I}}\right\}$
1 Initialization:2 Randomly select the feature vectors of I clients as the initial cluster centers.3 Initialize the clustering results, with each cluster initially empty.4 for iteration count t=1 to κ do5 Initialize the cluster list, with each cluster empty.6 for client n = 1, 2, …, N do7 Calculate the distance between the client and each cluster center:$8\;\tilde{d(n,m)}=\sqrt{{\left(\tilde{\left|D_{n}^{k}\right|}-\tilde{\left|D_{m}^{k}\right|}\right)}^{2}+{\left(\tilde{T_{n}^{k}}-\tilde{T_{m}^{k}}\right)}^{2}+{\left(\tilde{E_{n}^{k}}-\tilde{E_{m}^{k}}\right)}^{2}}$9 end for10 Assign the client to the nearest cluster:$11\;\tilde{d_{\min}(n,i)}\in \Lambda$$12\;\mathrm{for}\;\mathrm{each}\;\mathrm{cluster}\;C_{i}$ do13 Update the cluster center as the mean of the feature vectors of all clients in the cluster: $\tilde{\mu_{i}^{\left(t+1\right)}}=\frac{1}{\tilde{\left|C_{i}^{\left(t\right)}\right|}}\sum_{n\in \tilde{C_{i}^{\left(t\right)}}}\tilde{x_{n}}$
14 if the change in cluster centers is less than the threshold
ε
15 Stop iteration16 end if      end for17 end for$18\;\mathrm{Return}\;\tilde{C}=\left\{\tilde{C_{1}},\tilde{C_{2}},\ldots ,\tilde{C_{i}},\ldots ,\tilde{C_{I}}\right\}$

### 3.2. Hierarchical Agglomerative Evaluation Strategy

**Figure 4:**
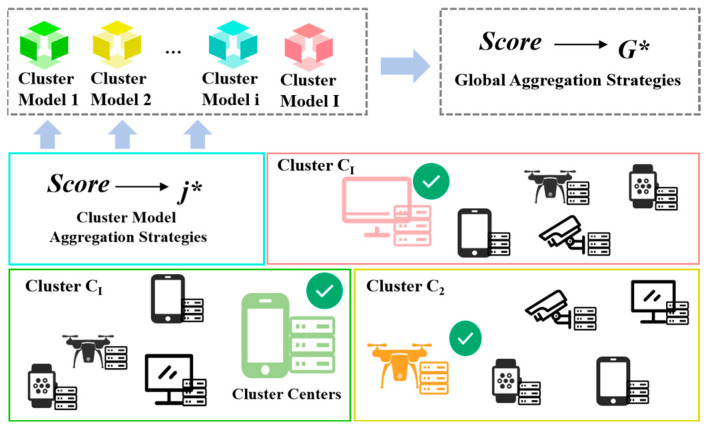
Hierarchical aggregation evaluation architecture.

In this section, a hierarchical aggregation evaluation strategy is proposed by leveraging the virtualized evaluation capabilities of the digital twin layer. As shown in Figure 4, after dynamic clustering, each cluster $\tilde{C_{i}}$ contains a specific set of clients $\tilde{O_{i}}$. A hierarchical aggregation strategy matrix is constructed in the DT layer, which encompasses cluster model aggregation strategies and global aggregation strategies. This matrix enables the DT to simulate the performance of various model combinations and aggregation algorithms in a virtual environment. Preaggregation evaluations are conducted on various combinations of client models within each cluster and the aggregated cluster models, which significantly reduces actual resource consumption and enables rapid iterative optimization of strategies under complex constraints.

The specific steps are as follows: First, within the digital twin layer, the clients within cluster $C_{i}$ are ranked. Since latency, energy consumption, and dataset size have different units and numerical scales, they are normalized before calculating the client efficiency score. The normalization is $\tilde{X}=\left(X-X_{\min}\right)/\left(X_{\max}-X_{\min}+\varepsilon \right)$, where X denotes latency, energy consumption, dataset size, or accuracy, and ε is a small positive constant used to avoid division by zero. Based on the normalized metrics, the client efficiency score and aggregation strategy score are calculated in the DT layer.

Since latency and energy consumption are undesirable cost terms, while dataset size reflects the potential data contribution of a client, the client efficiency score is formulated as a benefit–cost function:

(17) $${\tilde{M}}_{n}^{i}=-\psi_{1}{\tilde{T}}_{n}^{k}-\psi_{2}{\tilde{E}}_{n}^{k}+\psi_{3}{\tilde{D}}_{n}^{k}$$

where ${\tilde{T}}_{n}^{k},{\tilde{E}}_{n}^{k},{\tilde{D}}_{n}^{k}$ denote the normalized latency, energy consumption, and dataset size of client n in training round k, respectively. The coefficients $\psi_{1},\psi_{2}$ and $\psi_{3}$ are non-negative and satisfy $\psi_{1}+\psi_{2}+\psi_{3}=1$. A larger ${\tilde{M}}_{n}^{i}$ indicates that the client has a better trade-off between data contribution and resource cost. Therefore, clients are sorted in descending order according to ${\tilde{M}}_{n}^{i}$, and the top-ranked clients are selected as candidate participants for intra-cluster aggregation. In the experiments, $\psi_{1},\psi_{2}$ and $\psi_{3}$ are set to 1/3, 1/3, and 1/3, respectively, unless otherwise specified.

It should be noted that treating dataset size as a benefit term does not ignore its computation cost. A larger local dataset may provide more training samples and thus contribute more to global model training. However, the additional computation and communication overhead caused by a larger dataset is already reflected in the normalized latency and energy terms. Therefore, the client efficiency score balances the potential data contribution of larger datasets against their corresponding resource costs, rather than simply favoring clients with more data.

Next, an aggregation strategy matrix is constructed on the basis of the client list $\tilde{O}$ and the model aggregation algorithm space $\tilde{P}$. Each element in the matrix represents an aggregation strategy. The aggregation strategy matrix $\tilde{J_{O\times P}}$ is expressed as follows:

(18) $$\tilde{J_{O\times P}}=\left(\begin{array}{ccc} j_{11} & \ldots & j_{1P}\\ \vdots & \ddots & \vdots \\ j_{O1} & \ldots & j_{OP} \end{array}\right),$$

where $\tilde{O}$ denotes the list of clients that participate in aggregation within a cluster, $j_{o\times p}$ represents the aggregation strategy, and $\tilde{P}$ denotes the algorithm within the model aggregation algorithm space. The model aggregation algorithm space that is defined in this study includes four model aggregation algorithms, namely, simple averaging, weighted averaging, model performance-weighted, and model diversity-weighted, which are represented as follows:

(19) $$P=\left\{P_{1},P_{2},P_{3},P_{4}\right\},$$

where $P_{1}$ denotes the simple averaging algorithm, $P_{2}$ denotes the weighted averaging algorithm, $P_{3}$ denotes the model performance weighting algorithm, and $P_{4}$ denotes the model diversity weighting algorithm.

Second, because the optimization objective of this study is to select a subset of clients to participate in aggregation and the model aggregation method in each round to minimize the total latency and total energy consumption after K rounds of iterative training, the iterative latency $\tilde{T_{i}^{k}\left(j_{op}\right)}$, iterative energy consumption $\tilde{E_{i}^{k}\left(j_{op}\right)}$, and accuracy $\tilde{\vartheta_{i}}$ of the aggregated model on the validation set are chosen as metrics for evaluating the performance of the aggregation strategy.

Before actual server-side aggregation, the DT layer virtually evaluates each candidate aggregation strategy. A candidate strategy consists of a selected client subset and a specific aggregation algorithm. In the current implementation, the DT layer does not train an additional regression model or surrogate model for performance prediction. Instead, it estimates the latency and energy consumption of each candidate strategy according to the communication and computation models in Section 2, the DT-maintained client state profiles, and historical observations from previous completed rounds. Specifically, the estimated latency is obtained from the estimated latency of the selected clients and the estimated server-side aggregation latency. The estimated energy consumption is obtained from the estimated energy consumption of the selected clients and the server-side aggregation energy, which is calculated according to the server power and the estimated aggregation latency.

For model accuracy, the DT layer does not predict accuracy from client features. Instead, it performs virtual aggregation using the local model parameters mapped from the selected clients and the aggregation algorithm specified by the candidate strategy. The virtually aggregated model is then evaluated on the validation set maintained in the DT layer, and the obtained validation accuracy is used as the estimated accuracy of this candidate strategy. Therefore, the accuracy term is obtained by direct validation of the virtually aggregated model in the DT layer, rather than by regression-based prediction. In this way, the DT layer can compare different candidate strategies in terms of estimated latency, estimated energy consumption, and validation accuracy before the selected strategy is executed in the physical FL process.

For each candidate aggregation strategy, latency and energy consumption are regarded as cost terms, whereas model accuracy is regarded as a benefit term. Therefore, the aggregation strategy score is designed as a weighted cost–benefit function. Before calculating the score, latency, energy consumption, and model accuracy are normalized so that different metrics can be compared under the same scale. The evaluation formula for the aggregation strategy is as follows:

(20) $$Score\left(j_{op}^{i}\right)=\alpha_{1}\tilde{T}\left(j_{op}^{i}\right)+\alpha_{2}\tilde{E}\left(j_{op}^{i}\right)-\alpha_{3}\vartheta \left(j_{op}^{i}\right),$$

where $\tilde{T}\left(j_{op}^{i}\right),\tilde{E}\left(j_{op}^{i}\right),\vartheta \left(j_{op}^{i}\right)$ denote the normalized estimated latency, energy consumption, and validation accuracy under strategy $j_{op}^{i}$, respectively. The coefficients $\alpha_{1},\alpha_{2}$ and $\alpha_{3}$ are non-negative and satisfy $\alpha_{1}+\alpha_{2}+\alpha_{3}=1$. Since latency and energy consumption should be minimized, they are assigned positive signs. Since accuracy should be maximized, it is assigned a negative sign. Thus, a lower score indicates a better aggregation strategy. In the experiments, $\alpha_{1},\alpha_{2}$ and $\alpha_{3}$ are set to 1/3, 1/3, and 1/3, respectively, unless otherwise specified.

Next, the aggregation effects of various combinations are compared, and the strategy with the lowest evaluation score is selected as the optimal strategy $\tilde{J_{i}^{*}}$ for the corresponding cluster. This strategy includes the list of clients that participate in aggregation and the model aggregation method. On the basis of this aggregation strategy, each cluster performs intra-cluster aggregation to obtain the cluster aggregation model $\tilde{\omega_{i}}$.

Next, an aggregation strategy matrix $\tilde{G_{I\times P}}$ is constructed for all cluster aggregation models $\tilde{\omega_{i}}$. Experiments with various combinations of cluster models and aggregation methods are performed to evaluate the performance of the aggregated models. The aggregation results of various combinations are compared, and the strategy $\tilde{g_{i\times p}}$ with the lowest computational evaluation score is selected as the optimal global model aggregation strategy $\tilde{G^{*}}$. This strategy includes the list of cluster models that participate in global aggregation and the aggregation method.

Finally, the DT layer transmits the optimal cluster aggregation strategy $\tilde{J_{i}^{*}}$ and the optimal global aggregation strategy $\tilde{G^{*}}$ to the edge server. The server performs aggregation at the physical layer: First, it aggregates to obtain the cluster model $\omega_{i}$ on the basis of the optimal cluster aggregation strategy $\tilde{J_{i}^{*}}$; then, it performs aggregation to obtain the global model $\omega^{k}$ on the basis of the optimal global aggregation strategy $\tilde{G^{*}}$. During global aggregation, personalized client aggregation strategies are employed for cluster model aggregation. Clients and aggregation methods are dynamically selected to minimize the total latency and energy consumption of the cluster model aggregation. Second, when global aggregation is performed on the cluster models, the preaggregation strategy is reapplied to determine the cluster models that participate in global aggregation and the global aggregation method, thereby minimizing the total latency and total energy consumption of the global model aggregation process.

## 4. Simulation Analysis

In this study, we employ Python to simulate the FL system and conduct extensive simulation experiments to evaluate the effectiveness of our proposed algorithm. We first detail the experimental setup, then present the results obtained for the prototype system and the simulation environment, and finally briefly summarize these findings.

### 4.1. Simulation Experimental Setup

In this paper, we propose a federated learning dynamic aggregation scheme that is based on digital twin technology. An experimental federated learning architecture that comprises one edge server and 30 clients is constructed for validation. The experimental environment is built using Python 3.8 by primarily relying on libraries such as PyTorch 1.9.0, torch vision 0.10.0, NumPy 1.19.5, Matplotlib 3.4.2, and Scikit-learn 0.24.2, which provide robust technical support for the experiments. A digital twin layer is constructed on the server side to precisely map the physical layer, which creates a corresponding digital twin for each physical client. These twins maintain high logical synchronization with their physical counterparts. Through real-time updates and maintenance, the twins accurately reflect key characteristics and status information of physical clients, such as dataset size, total communication latency, and overall energy consumption.

In this study, a fixed set of weights is used throughout the experiments to ensure a consistent comparison among different algorithms. Specifically, $\alpha_{1},\alpha_{2}$ and $\alpha_{3}$ are set to 1/3, 1/3, and 1/3, respectively; $\psi_{1},\psi_{2}$ and $\psi_{3}$ are set to 1/3, 1/3, and 1/3, respectively; and ξ is set to 0.5. These settings provide a balanced operating point among latency, energy consumption, and model accuracy. The weighting factor ξ controls the trade-off between latency and energy consumption in the optimization objective. A larger ξ gives higher priority to latency reduction, whereas a smaller ξ emphasizes energy saving. The coefficients $\alpha_{1},\alpha_{2}$ and $\alpha_{3}$ control the strategy evaluation preference in the DT layer. Increasing $\alpha_{1}$ tends to select aggregation strategies with lower estimated latency, increasing $\alpha_{2}$ favors strategies with lower estimated energy consumption, and increasing $\alpha_{3}$ gives more importance to validation accuracy. Similarly, the coefficients $\psi_{1},\psi_{2}$ and $\psi_{3}$ affect client ranking within each cluster. Larger $\psi_{1}$ and $\psi_{2}$ penalize clients with higher estimated latency and energy consumption, while a larger $\psi_{3}$ favors clients with larger local datasets and potentially higher data contribution. Therefore, different weight settings can lead to different latency-energy-accuracy trade-offs. In latency-sensitive applications, larger ξ and $\alpha_{1}$ can be used to reduce training latency. In energy-constrained scenarios, smaller ξ and larger $\alpha_{2}$ can be adopted to reduce energy consumption. In accuracy-oriented applications, larger $\alpha_{3}$ and $\psi_{3}$ can be used to give higher priority to model accuracy and data contribution. A systematic sensitivity analysis of these weighting coefficients is beyond the scope of the current work and will be conducted in future work.

For experimentation, two public datasets are employed: CIFAR-10 and MNIST. The CIFAR-10 dataset comprises 60,000 32 × 32-pixel color images that span 10 distinct categories—including aircraft, automobiles, birds, and animals—with 50,000 images in the training set and 10,000 in the test set. For CIFAR-10, a convolutional neural network (CNN) is designed, which comprises two convolutional layers and one fully connected layer. These layers extract local image features and integrate outputs for final classification. The MNIST dataset comprises 70,000 grayscale handwritten digit images that measure 28 × 28 pixels, which are also categorized into 10 classes (handwritten digits 0 through 9). This dataset is similarly divided into a training set and a test set of 60,000 and 10,000 images, respectively. For the MNIST dataset, a multilayer perceptron (MLP) is designed. This model comprises an input layer, a hidden layer, and an output layer. The input layer receives pixel values as feature vectors, the hidden layer captures complex patterns through nonlinear transformations, and the output layer classifies the input images to identify the corresponding digit category. To improve reproducibility, the experimental settings are further specified in Table 3. The MNIST and CIFAR-10 training samples are distributed among 30 clients under a non-IID label-skew setting. Specifically, each client holds samples from two randomly selected classes, and the local dataset size varies across clients to simulate data heterogeneity. In addition to data heterogeneity, system heterogeneity is considered from two aspects: computing capability and wireless communication conditions. The local CPU frequency of each client is randomly selected from 0.5 GHz to 2.0 GHz, and the distance between each client and the edge server is randomly generated within 50 m to 500 m. The wireless channel follows Rayleigh fading with distance-dependent path loss. The same data partition, client heterogeneity settings, wireless parameters, and training hyperparameters are used for all compared algorithms to ensure a fair comparison. The number of clusters is determined by the silhouette coefficient method within the candidate range of 2 to 15. In the experiments, the selected number of clusters I is set as 5 for both the MNIST and CIFAR-10 datasets.

As shown in Table 3, the non-IID data distribution is generated by a label-skew partition. Each client is assigned samples from two randomly selected classes, and the number of samples varies across clients. This setting reflects both statistical heterogeneity and data-size heterogeneity among clients. The communication parameters are configured according to the wireless communication model described in Section 2.1. Specifically, the channel gain is determined by Rayleigh fading and distance-dependent path loss. The computation-related parameters are configured according to the computational model described in Section 2.2. A fixed random seed is used for data partitioning, client parameter generation, and model initialization to ensure reproducibility. The reported curves are obtained under this fixed-seed setting. All compared algorithms are evaluated under the same experimental configuration. A systematic repeated-run evaluation with multiple random seeds will be considered in future work to further analyze statistical variability.

The proposed DT-FL algorithm is evaluated against three baseline algorithms in the experiments. (1) The first baseline is the classic FL algorithm FedAvg [1], which updates the global model by simply averaging all the client models. (2) The second baseline is the Non-DT algorithm, which removes the DT layer from DT-FL. In this baseline, client clustering and preaggregation evaluation are directly performed by the physical server using the currently available client information, without DT-based state mapping and virtual evaluation. (3) The third baseline is the Non-clustering algorithm, which removes the dynamic client clustering process from DT-FL. In this baseline, all clients are treated as a single group, and the DT layer only evaluates the four aggregation modes defined in the model aggregation algorithm space. It does not exhaustively enumerate all possible client subsets. The aggregation mode with the best evaluation score is selected for global aggregation in the physical layer. Therefore, the search space of the Non-clustering baseline is limited to the four candidate aggregation modes, rather than $2^{N}$ client subsets. These baselines can also be interpreted as component-level ablation variants. DT-FL is the full version with both DT-based virtual evaluation and dynamic client clustering. Non-clustering removes the dynamic clustering module and is used to evaluate the contribution of client clustering. Non-DT removes the DT layer and is used to evaluate the contribution of DT-based state mapping and virtual evaluation. FedAvg serves as the classical FL baseline without DT-based evaluation, dynamic clustering, or dynamic aggregation strategy selection.

In the experiments, the FedAvg, Non-DT, and Non-clustering algorithms maintain configurations that are identical to those of DT-FL, including identical client counts, training rounds, and local training rounds. To clarify algorithm performance comparisons, the average latency, energy consumption, and accuracy data of the experiments are compared every five rounds, after which their evolution over iterations is observed. The selected baselines are designed to evaluate the contribution of different components of the proposed framework. FedAvg is used as the classical FL reference. The Non-DT baseline is used to evaluate the benefit of introducing the DT layer, while the Non-clustering baseline is used to evaluate the contribution of client clustering. Therefore, these baselines serve as component-level comparisons for the proposed DT-based preaggregation evaluation and clustering mechanisms. FedProx, SCAFFOLD, and representative clustered FL methods are strong FL baselines for handling statistical heterogeneity and client drift. However, these methods focus on different aspects of FL optimization. FedProx and SCAFFOLD mainly improve local training stability under non-IID data by modifying the local objective or introducing correction terms. Representative clustered FL methods mainly group clients according to data or model similarity to improve aggregation under heterogeneous distributions. In contrast, the proposed DT-FL framework focuses on DT-based preaggregation evaluation and dynamic aggregation strategy selection before physical aggregation. Specifically, DT-FL uses the DT layer to virtually evaluate candidate aggregation strategies in terms of latency, energy consumption, and validation accuracy, and then selects the most suitable strategy for physical-layer execution. Therefore, these advanced FL methods are conceptually complementary to the proposed framework and can potentially be integrated into the DT layer as alternative local training or aggregation modules. A more comprehensive comparison with representative advanced FL baselines will be conducted in future work.

### 4.2. Experimental Results and Discussion

**Figure 5:**
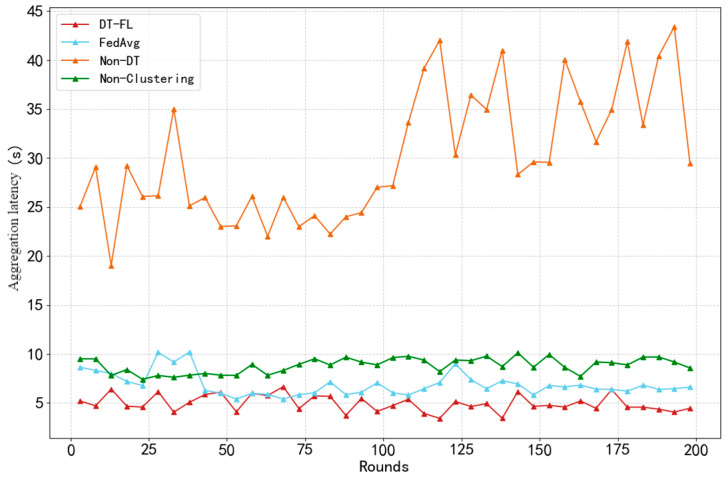
Aggregation latency comparison of the DT-FL and three baseline algorithms on the MNIST dataset.

**Figure 6:**
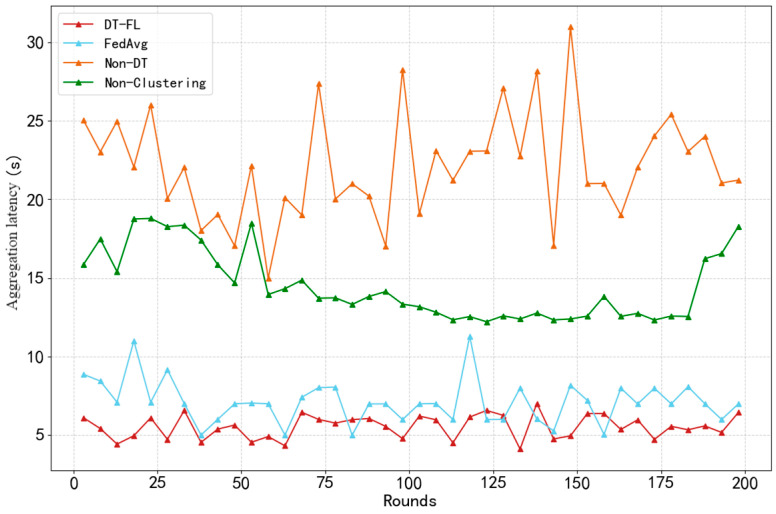
Aggregation latency comparison between the DT-FL and three baseline algorithms on the CIFAR-10 dataset.

Figure 5 and Figure 6 illustrate the evolution of the aggregation latency over training iterations for DT-FL and the three baseline algorithms on the MNIST and CIFAR-10 datasets, respectively. The aggregation latency for the DT-FL and Non-clustering algorithms includes both the physical-layer aggregation latency and DT-layer preaggregation latency. The experimental results demonstrate that the DT-FL algorithm achieves significantly lower aggregation latency than the FedAvg, Non-DT, and Non-clustering algorithms do and that the performance is more stable, with markedly reduced latency fluctuations. This stems from DT-FL’s incorporation of optimal aggregation strategies from the DT layer during physical-layer aggregation. This approach ensures that participating clients not only exhibit optimal performance but also adopt the lowest-latency aggregation method, thereby substantially increasing the overall aggregation efficiency. Although the DT-FL algorithm introduces an additional step of preaggregation evaluation in the DT layer during the aggregation phase, this step does not significantly increase the overall aggregation latency. This is because the DT layer relies primarily on the powerful computational capabilities of servers for its construction. This demonstrates that the DT-FL algorithm cleverly balances the relationship between additional functionality and system efficiency in its design, thus avoiding performance degradation caused by added features.

Furthermore, compared with the Non-clustering algorithm, DT-FL employs a more efficient client clustering strategy. Within DT-FL, clients are first clustered on the basis of their feature vectors to form multiple clusters. For the aggregation process for each cluster, the algorithm adopts the optimal aggregation strategy with minimal latency. These strategies include carefully selected lists of participating clients and the most suitable aggregation methods. Furthermore, the DT-FL algorithm does not involve all the clients in aggregation. Instead, it selects the most performant clients to participate, thereby reducing unnecessary communication and computational overhead. This strategy not only decreases the aggregation latency but also improves the system’s overall efficiency and resource utilization.

**Figure 7:**
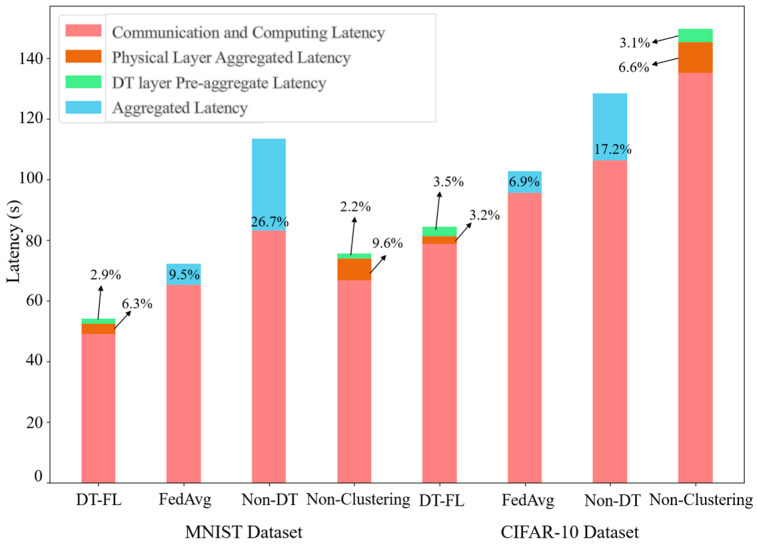
Comparison of the aggregation latency of the four algorithms on two datasets.

In this study, the communication and computational latency of DT-FL and the Non-clustering algorithm, the physical-layer aggregation latency, the DT-layer preaggregation latency, and the communication and computational latency and aggregation latency of the FedAvg and Non-DT algorithms are averaged. Then, the proportion of the aggregation latency relative to the total latency is calculated for each of the three algorithms across different datasets, and the results are shown in Figure 7. The experimental data indicate that DT-FL exhibits an extremely low proportion of preaggregation latency at the DT layer, which is consistently maintained below 4% of the total latency. This finding demonstrates that despite the introduction of the twin-layer mechanism, its effect on the overall latency is relatively small in the simulated setting, and it does not dominate the total training latency in the simulated setting. Moreover, while maintaining a low proportion of preaggregation latency, the DT-FL algorithm shows better performance in terms of the proportion of the physical-layer aggregation latency. Its physical-layer aggregation latency proportion is consistently lower than the corresponding metrics of the three baseline algorithms, namely, the FedAvg, Non-DT, and Non-clustering algorithms. These results support the effectiveness of the twin-layer preaggregation strategy of the DT-FL algorithm, which significantly reduces the aggregation latency and improves the operational efficiency of FL systems. In contrast, the FedAvg, Non-DT, and Non-clustering algorithms lacking dynamic optimization mechanisms perform poorly in terms of both the aggregation latency ratio and total latency. These algorithms cannot match the ability of the DT-FL algorithm to pre-evaluate and optimize aggregation strategies at the DT layer, thereby reducing latency while ensuring system efficiency. Consequently, they exhibit relatively high aggregation latency ratios and total latency, which limits their application potential in highly efficient FL systems.

It should also be noted that maintaining and updating the DT layer introduces additional computational overhead. This overhead mainly comes from client state synchronization, DT profile updating, client clustering, candidate strategy evaluation, and virtual aggregation on the validation set. In the current simulation setting, the DT-related preaggregation latency accounts for a relatively small proportion of the total latency, as shown in Figure 7. This indicates that the overhead of the DT layer does not dominate the total training cost under the considered scale. However, in large-scale FL systems with a much larger number of clients, the computational cost of clustering and candidate strategy evaluation may increase. Therefore, scalable DT maintenance, lightweight strategy evaluation, and efficient candidate pruning are important issues for practical deployment. These aspects will be further investigated in future work.

**Figure 8:**
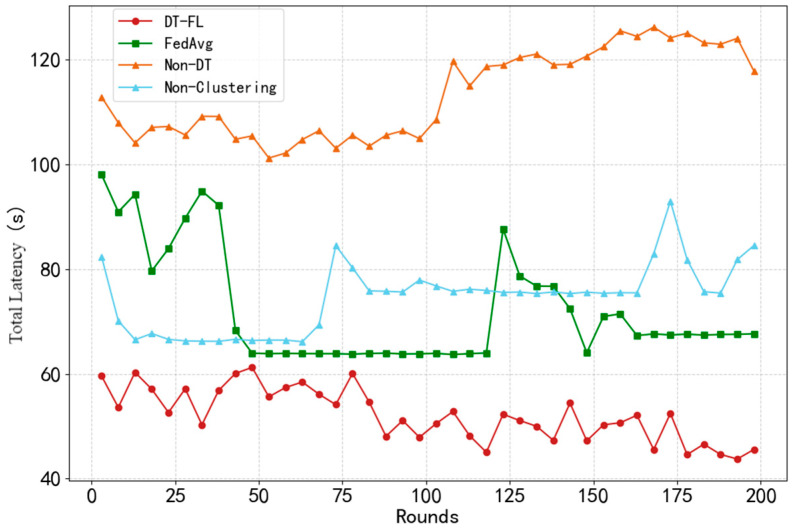
Total latency comparison between DT-FL and three baseline algorithms on the MNIST dataset.

**Figure 9:**
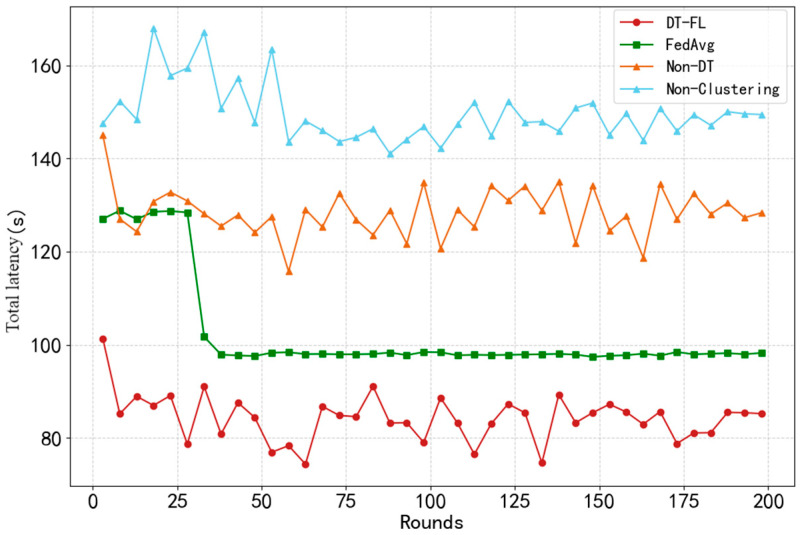
Total latency comparison between DT-FL and three baseline algorithms on the CIFAR-10 dataset.

As shown in Figure 8 and Figure 9, the proposed DT-FL algorithm significantly outperforms the FedAvg, Non-DT, and Non-clustering baseline algorithms on both the MNIST and CIFAR-10 datasets. On the MNIST dataset, compared with the three baselines, DT-FL reduces the total latency by approximately 22%, 53%, and 42%, respectively. On CIFAR-10, the reductions reach approximately 20%, 38%, and 47%, respectively. This advantage stems from the use of K-means clustering by DT-FL to efficiently group clients on the basis of feature similarity. Following clustering, the algorithm performs hierarchical model aggregation both within and between clusters, which reduces redundant communication overhead and significantly increases the overall efficiency. In contrast, the FedAvg algorithm employs a full-client participation aggregation mechanism that requires all the clients to transmit and update the model parameters during each training iteration. This large-scale data transfer inevitably creates communication bottlenecks, thus severely limiting potential efficiency gains. In contrast, the Non-DT algorithm lacks a DT layer, which forces its client dynamic clustering and preaggregation strategy evaluation to occur at the physical layer. This increases the overall computational and communication overhead, thus leading to significantly higher latency.

**Figure 10:**
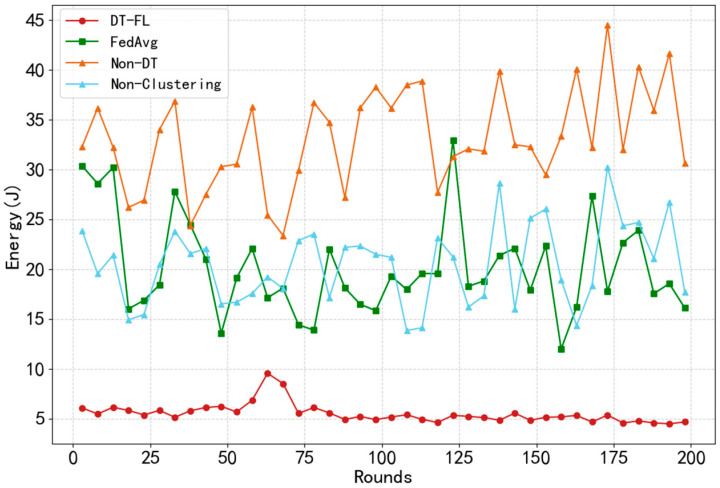
Total energy consumption comparison between DT-FL and three baseline algorithms on the MNIST dataset.

**Figure 11:**
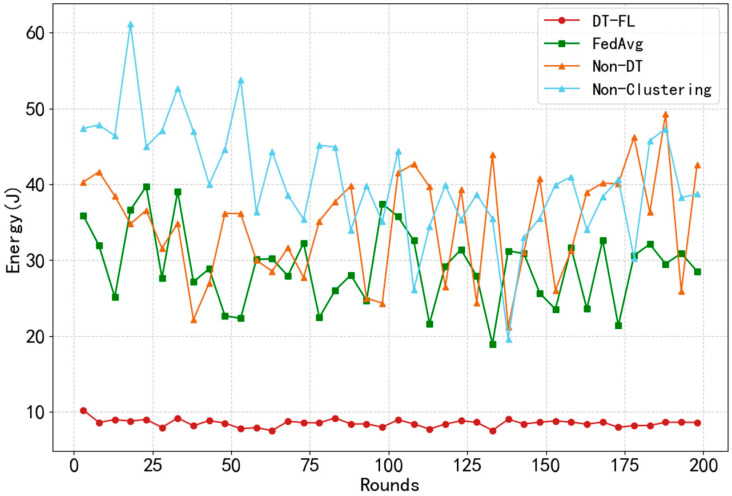
Total energy consumption comparison between DT-FL and three baseline algorithms on the CIFAR-10 dataset.

Comparisons of total energy consumption among the DT-FL, FedAvg, Non-DT, and Non-clustering algorithms on the MNIST and CIFAR-10 datasets are shown in Figure 10 and Figure 11, respectively. Compared with the other three baseline algorithms, DT-FL consistently achieves lower total energy consumption. Compared with the baselines, DT-FL achieves up to 77% and 83% total energy optimization on the MNIST and CIFAR-10 datasets, respectively. Additionally, DT-FL results in a smoother total energy consumption curve, which indicates superior stability and convergence during training. This advantage stems from the dual optimization mechanism of DT-FL. First, by leveraging DT technology to construct a pre-aggregated model, DT-FL dynamically simulates and evaluates client participation. This enables the algorithm to identify clients that contribute minimally or inefficiently to model training and reduce their involvement. This process increases the overall training efficiency while effectively lowering the energy consumption. In addition, during intra-cluster and inter-cluster aggregation, the DT-FL algorithm dynamically selects different aggregation methods on the basis of client data and status information. It adaptively adjusts weights according to client feature vectors to prevent excessive participation from inefficient clients, thereby reducing unnecessary computational and communication overhead.

Furthermore, the performance of the DT-FL algorithm is simulated under various aggregation strategies using DT technology. The algorithm evaluates the effects of various strategies on energy consumption without actually executing aggregation. This enables the algorithm to preemptively select the optimal aggregation strategy, thus avoiding the increased energy consumption caused by frequent strategy trials during actual aggregation. In contrast, Non-DT algorithms require dynamic client clustering and preaggregation evaluation at the physical layer, which leads to a significant increase in total energy consumption. By performing these evaluations in the DT layer, the DT-FL algorithm effectively reduces the computational and communication overhead in the physical layer, thus achieving substantial energy savings.

**Figure 12:**
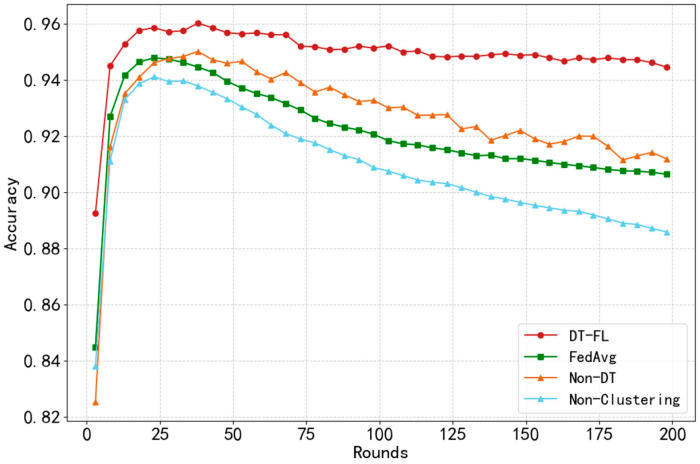
Model accuracy comparison between DT-FL and three baseline algorithms on the MNIST dataset.

**Figure 13:**
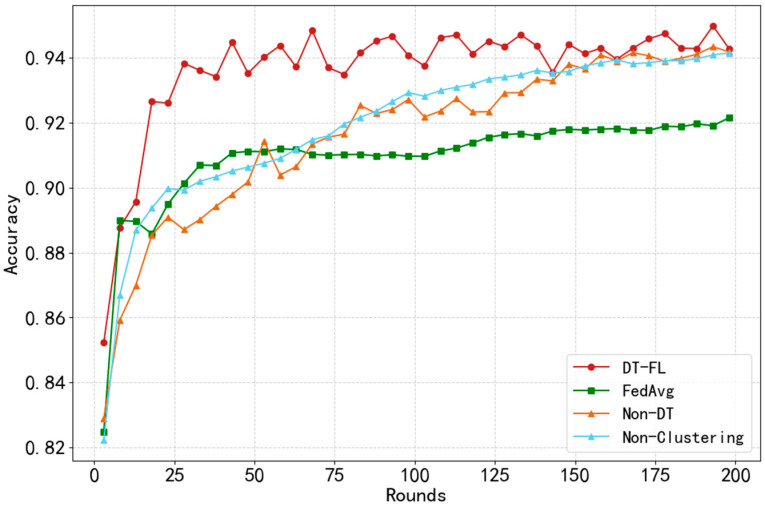
Model accuracy comparison between DT-FL and three baseline algorithms on the CIFAR-10 dataset.

Comparisons of model accuracy among the DT-FL, FedAvg, Non-DT, and Non-clustering algorithms on the MNIST and CIFAR-10 datasets are shown in Figure 12 and Figure 13, respectively. The DT-FL algorithm demonstrates significant advantages in terms of both faster convergence and higher accuracy across both datasets. As shown in Figure 11, on the MNIST dataset, DT-FL exhibits rapid convergence during the early training phase (0–20 epochs), with the model accuracy increasing sharply before stabilizing near 0.95. In contrast, the final accuracies of the three baseline algorithms all remain below 0.92. This significant difference stems primarily from the dynamic cluster aggregation mechanism of the DT-FL algorithm. By adaptively clustering client features (such as dataset size, total latency, and total energy consumption), the algorithm fully considers the distribution of data within each cluster, thereby enabling more reasonable weight allocation during aggregation. This mechanism enables clusters with similar data distributions to collaborate more effectively, thereby increasing model accuracy. On the CIFAR-10 dataset, as shown in Figure 12, the DT-FL algorithm similarly demonstrates rapid convergence and high accuracy. Its final accuracy significantly surpasses that of the other baseline algorithms, which validates the stability and effectiveness of the DT-FL algorithm across diverse datasets.

Furthermore, the Non-DT algorithm outperforms the FedAvg and Non-clustering algorithms in terms of convergence speed and final accuracy. This advantage stems primarily from its ability to perform dynamic clustering and preaggregation evaluation in the physical layer. This approach enables the server to execute optimal aggregation strategies, thereby achieving the best possible results for global aggregation. However, this mechanism introduces additional computational and communication overhead, thus significantly increasing the overall latency of the Non-DT algorithm and consequently impacting its efficiency in practical applications. In contrast, the DT-FL algorithm avoids frequent dynamic clustering and preaggregation operations in the physical layer by performing preaggregation evaluation in the DT layer. This significantly reduces the computational and communication overhead, which enables DT-FL to maintain low latency while ensuring high accuracy and rapid convergence. These results demonstrate its potential advantage in federated learning.

Although the current figures are reported under a fixed-seed setting, all compared algorithms are evaluated under the same data partition, client heterogeneity settings, wireless communication parameters, and training hyperparameters. Therefore, the comparisons reflect the performance differences under identical experimental configurations. We agree that error bars or confidence intervals can further improve the statistical presentation of the results. We also acknowledge that multi-seed experiments with standard deviations would provide stronger statistical evidence for the robustness of DT-FL. Therefore, repeated experiments with multiple random seeds will be prioritized in our future experimental evaluation to report confidence intervals and further assess statistical robustness.

## 5. Conclusions

This paper proposed a digital twin-based dynamic aggregation algorithm for federated learning to address the heterogeneous training challenges of heterogeneous sensor terminals with massive computing capabilities in next-generation IoT perception networks. This method leverages DTs and machine learning for global model preaggregation evaluation to maximize training accuracy while minimizing latency and energy consumption. First, we applied a K-means-based client-adaptive clustering method. The optimal number of clusters is determined by using the silhouette coefficient method, and then the clustering algorithm is executed to construct an efficient client cluster topology. Then, we established an aggregation strategy matrix for different clusters and applied a hierarchical aggregation evaluation strategy. The performance metrics include the average total latency and average total energy consumption of the clients within a cluster, along with the accuracy of the aggregated model on the validation set. Additionally, we designed a dual evaluation mechanism to assess both the intra-cluster model preaggregation strategies and the inter-cluster preaggregation strategies, thereby improving the overall performance of FL. Simulation analysis on the MNIST and CIFAR-10 datasets demonstrates that this method not only accelerates model convergence and improves accuracy but also significantly reduces training latency and energy consumption costs.

## Figures and Tables

**Table 1:** Qualitative comparison with related FL aggregation methods.

Method	Decision Basis	Clustering	Hierarchical Aggregation	DT-Based Evaluation	Dynamic Strategy Selection
FedAvg-based methods [1]	Averaging rule	No	No	No	No
Weighted aggregation methods [11,15,17]	Model/data quality	No	No	No	Partial
DT-assisted FL methods [27,28,29,30]	DT-modeled state	Partial	Partial	Partial	Partial
Client selection methods [31]	Client state	No	No	No	Partial
Clustered FL methods [32]	Client similarity	Yes	Partial	No	Partial
Hierarchical FL methods [16,32]	Network or model hierarchy	Partial	Yes	No	Partial
Proposed DT-FL	DT-estimated strategy performance	Yes	Yes	Yes	Yes

**Table 2:** Client Cluster Center Euclidean Distance Matrix.

	$\mu_{1}$	…	$\mu_{i}$	…	$\mu_{I}$
1	$\tilde{d(1,1)}$		$\tilde{d(1,i)}$		$\tilde{d(1,I)}$
2	$\tilde{d(2,1)}$		$\tilde{d(2,i)}$		$\tilde{d(2,I)}$
**⋮**	**⋮**		**⋮**		**⋮**
N	$\tilde{d(N,1)}$	**…**	$\tilde{d(N,i)}$	**…**	$\tilde{d(N,I)}$

**Table 3:** Experimental Simulation Parameter Settings.

Parameter Name	Parameter Value	Description
Number of clients	30	Number of simulated FL clients
Training Rounds (num_rounds)	200	The total number of global training rounds, i.e., the number of times the global model is updated
Local Epochs (epochs)	5	The number of local training epochs each client performs in every global training round
Learning Rate (lr)	0.01	Parameter used to control the step size for weight updates
Batch Size (batch_size)	10	The number of samples processed together in a single training step before the model is updated.
$\mathrm{Local}\;\mathrm{dataset}\;D_{n}$	Non-IID label-skew	Local dataset owned by client n
Non-IID setting	2 classes/client	Each client holds samples from two randomly selected classes
Client dataset size	800–2000 samples	Local dataset size varies across clients
Total uplink bandwidth B	10 MHz	Total wireless bandwidth provided by the edge server
$\mathrm{Client}\;\mathrm{transmit}\;\mathrm{power}\;p_{u}^{k}$	0.1 W	Transmission power for uploading local model parameters
$\mathrm{Server}\;\mathrm{transmit}\;\mathrm{power}\;p_{n}^{k}$	1 W	Transmission power for broadcasting the global model
$\mathrm{Noise}\;\mathrm{power}\;N_{0}$	−104 dBm	Gaussian noise power
Client-server distance $d_{n}$	50–500 m	Distance between client n and the edge server
$\mathrm{CPU}\;\mathrm{cycles}\;\mathrm{per}\;\mathrm{sample}\;c_{n}$	$10^{5}$ cycles/sample	CPU cycles required for processing one data sample
$\mathrm{Rayleigh}\;\mathrm{fading}\;\mathrm{parameter}\;o_{n}$	1	Small-scale fading parameter
$\mathrm{Channel}\;\mathrm{gain}\;h_{n}^{k},h_{u}^{k}$	$o_{n}d_{n}^{-2}$	Channel gain between client n and the edge server
$\mathrm{Client}\;\mathrm{CPU}\;\mathrm{frequency}\;f^{k}$	0.5–2.0 GHz	Local CPU frequency allocated to client computation
$\mathrm{Server}\;\mathrm{power}\;P_{s}$	50 W	Power consumption during DT evaluation and server aggregation

## Data Availability

Data are contained within the article.

## References

[1] McMahan B., Moore E., Ramage D., Hampson S., Agüera y Arcas B. Communication-Efficient Learning of Deep Networks from Decentralized Data. Proceedings of the 20th International Conference on Artificial Intelligence and Statistics.

[2] Chen M., Zhao L., Chen J., Wei X., Guizani M. (2023). Modal-Aware Resource Allocation for Cross-Modal Collaborative Communication in IIoT. IEEE Internet Things J..

[3] Kairouz P., McMahan H.B., Avent B., Bellet A., Bennis M., Bhagoji A.N., Bonawitz K., Charles Z., Cormode G., Cummings R. (2021). Advances and Open Problems in Federated Learning. Found. Trends Mach. Learn..

[4] Zhou S., Wang Y., Wang L., Chen L., Wang Y. (2026). FedDG: An Efficient Federated Learning Framework for IoT Communication Based on Dynamic Fusion and Gradient Compression. IEEE Trans. Ind. Inform..

[5] Shan F., Li S., Lu Y., Mao S., Wang X. (2025). SAFL-KCS: Federated Learning Methods Based on Client Selection and Semi-Asynchronous Communication. Clust. Comput..

[6] Li G., Chen M., Wei X., Qi T., Zhuang W. Computation Offloading With Reinforcement Learning in D2D-MEC Network. Proceedings of the 2020 International Wireless Communications and Mobile Computing (IWCMC).

[7] Jia N., Qu Z., Ye B., Wang Y., Hu S., Guo S. (2025). A Comprehensive Survey on Communication-Efficient Federated Learning in Mobile Edge Environments. IEEE Commun. Surv. Tutor..

[8] Xie R., Chen Z., Cao W., Wang H. (2026). Federated Self-Expanding Neural Network Learning Framework for Heterogeneous Devices. Expert Syst. Appl..

[9] Yao T., Li J., Liu J. (2026). FedAWR: Aggregation Optimization in Federated Learning with Adaptive Weights and Learning Rates. Future Internet.

[10] Peng Y.Z., Song Q.H., Wang R.Q., Yang X.Y., Liu Z.Q., Liu Z.J. (2024). A Tool Wear Condition Monitoring Method for Non-Specific Sensing Signals. Int. J. Mech. Sci..

[11] Bensiah O.A., Benaboud R. (2025). Addressing Data Heterogeneity in Federated Learning: The FedWAQ Approach to Weighted Aggregation Based on Data Quality. 3rd International Conference on Computer Science’s Complex Systems and Their Applications (ICCSA).

[12] Xiao J., Wang S. Data Aggregation Method for Privacy Protection in Federated Learning Environment. Proceedings of the 2023 3rd International Conference on Electronic Information Engineering and ComputerTechnology (EIECT).

[13] Xu X., Xu Y., Dou H., Chen M., Wang L. (2024). Federated KD-Assisted Image Semantic Communication in IoT Edge Learning. IEEE Internet Things J..

[14] Chen M., Wang C., He X., Zhu F., Wang L., Vasilakos A.V. (2025). Embodied Artificial Intelligence-Enabled Internet of Vehicles: Challenges and Solutions. IEEE Veh. Technol. Mag..

[15] Xie Y., Pei F., Shi M. FedCoef: An Adaptive Aggregation Algorithm Based on Coefficient of Variation for Federated Learning. Proceedings of the 14th Asian Control Conference.

[16] Zhang W., Zhou T., Lu Q., Yuan Y., Tolba A., Said W. (2024). FedSL: A Communication-Efficient Federated Learning with Split Layer Aggregation. IEEE Internet Things J..

[17] Ayoub E., Ahmad A., Abdelrahman A., Mohammad H., Ammar M. (2024). SimProx: A Similarity-Based Aggregation in Federated Learning with Client Weight Optimization. IEEE Open J. Commun. Soc..

[18] Li X., Chen Y., Wang Z., Zhang L. (2023). Adaptive Model Aggregation for Federated Learning: A Comprehensive Framework and Evaluation. IEEE Trans. Parallel Distrib. Syst..

[19] Hu X., Wen P., Xiao H., Wang W., Wong K.K. (2025). Maximizing Energy Charging for UAV-Assisted MEC Systems With SWIPT. IEEE Trans. Veh. Technol..

[20] Mehrad V., Kiana N., Terence D.T., Dongmei Z., George K. (2023). Digital Twin Placement for Minimum Application Request Delay with Data Age Targets. IEEE Internet Things J..

[21] Xiao H., Hu X., Zhang W., Wang W., Wong K.K., Yang K. (2025). Energy-Efficient STAR-RIS Enhanced UAV-Enabled MEC Networks with Bi-Directional Task Offloading. IEEE Trans. Wirel. Commun..

[22] Xiao H., Hu X., Wang W., Su Z., Wong K.K., Yang K. (2025). STAR-RIS and UAV Combination in MEC Networks: Simultaneous Task Offloading and Communications. IEEE Trans. Commun..

[23] Li J., Yang S.X. (2025). Digital Twins to Embodied Artificial Intelligence: Review and Perspective. Intell. Robot..

[24] Lu Q., Cheng L., Zhu D., Li M. (2026). Fault Prediction Method towards Gearbox Based on Digital Twin and Deep Transfer Learning. Eng. Res. Express.

[25] Zhang Y., Li J., Wang H. (2025). Towards Heterogeneity-Aware and Energy-Efficient Topology Optimization for Decentralized Federated Learning. arXiv.

[26] Linbo H., Mowei W., Liang Z., Lu Y., Chen Y. (2023). Digital Twin for Networking: A Data-Driven Performance Modeling Perspective. IEEE Netw..

[27] Lu Y., Huang X., Zhang K., Maharjan S., Zhang Y. (2021). Communication-Efficient Federated Learning for Digital Twin Edge Networks in Industrial IoT. IEEE Trans. Ind. Inform..

[28] Guo J., Liu Z., Tian S., Huang F., Li J., Li X., Igorevich K.K., Ma J. (2023). TFL-DT: A Trust Evaluation Scheme for Federated Learning in Digital Twin for Mobile Networks. IEEE J. Sel. Areas Commun..

[29] Tang Y., Wang K., Niyato D., Chen W., Karagiannidis G.K. (2025). Digital Twin-Assisted Federated Learning with Blockchain in Multi-Tier Computing Systems. IEEE Trans. Cogn. Commun. Netw..

[30] Sun W., Lei S., Wang L., Liu Z., Zhang Y. (2021). Adaptive Federated Learning and Digital Twin for Industrial Internet of Things. IEEE Trans. Ind. Inform..

[31] Yu L., Albelaihi R., Sun X., Ansari N., Devetsikiotis M. (2022). Jointly Optimizing Client Selection and Resource Management in Wireless Federated Learning for Internet of Things. IEEE Internet Things J..

[32] Wang Z., Xu H., Liu J., Xu Y., Huang H., Zhao Y. (2023). Accelerating Federated Learning with Cluster Construction and Hierarchical Aggregation. IEEE Trans. Mob. Comput..

[33] Wang Y., Fang J., Cheng Y., She H., Guo Y., Zheng G. (2024). Cooperative End-Edge-Cloud Computing and Resource Allocation for Digital Twin Enabled 6G Industrial IoT. IEEE J. Sel. Top. Signal Process..

[34] Zhang H., Yang Y., Shang B., Zhang P. (2022). Joint Resource Allocation and Multi-Part Collaborative Task Offloading in MEC Systems. IEEE Trans. Veh. Technol..

[35] Ikotun A.M., Ezugwu A.E., Abualigah L., Abuhaija B., Jia H. (2023). K-Means Clustering Algorithms: A Comprehensive Review, Variants Analysis, and Advances in the Era of Big Data. Inf. Sci..

